# Mesenchymal Stromal Cells: a Possible Reservoir for HIV-1?

**DOI:** 10.1007/s12015-021-10298-5

**Published:** 2022-01-01

**Authors:** K. Kallmeyer, M. A. Ryder, M. S. Pepper

**Affiliations:** 1 Department of Immunology, Institute for Cellular and Molecular Medicine, University of Pretoria, Pretoria, South Africa; 2 SAMRC Extramural Unit for Stem Cell Research and Therapy, Faculty of Health Sciences, University of Pretoria, Pretoria, South Africa

**Keywords:** Human immunodeficiency virus (HIV)-1, mesenchymal stromal/stem cells (MSCs), cellular reservoirs, receptors/co-receptors, HIV-1 proteins, HIV-1 latency, stem cells, infection, viral rebound

## Abstract

The introduction of antiretroviral therapy (ART) and highly active antiretroviral therapy (HAART) has transformed human immunodeficiency virus (HIV)-1 into a chronic, well-managed disease. However, these therapies do not eliminate all infected cells from the body despite suppressing viral load. Viral rebound is largely due to the presence of cellular reservoirs which support long-term persistence of HIV-1. A thorough understanding of the HIV-1 reservoir will facilitate the development of new strategies leading to its detection, reduction, and elimination, ultimately leading to curative therapies for HIV-1. Although immune cells derived from lymphoid and myeloid progenitors have been thoroughly studied as HIV-1 reservoirs, few studies have examined whether mesenchymal stromal/stem cells (MSCs) can assume this function. In this review, we evaluate published studies which have assessed whether MSCs contribute to the HIV-1 reservoir. MSCs have been found to express the receptors and co-receptors required for HIV-1 entry, albeit at levels of expression and receptor localisation that vary considerably between studies. Exposure to HIV-1 and HIV-1 proteins alters MSC properties *in vitro,* including their proliferation capacity and differentiation potential. However, *in vitro* and *in vivo* experiments investigating whether MSCs can become infected with and harbour latent integrated proviral DNA are lacking. In conclusion, MSCs appear to have the potential to contribute to the HIV-1 reservoir. However, further studies are needed using techniques such as those used to prove that cluster of differentiation (CD)4^+^ T cells constitute an HIV-1 reservoir before a reservoir function can definitively be ascribed to MSCs.

**Graphical abstract:**

MSCs may contribute to HIV-1 persistence *in vivo* in the vasculature, adipose tissue, and bone marrow by being a reservoir for latent HIV-1. To harbour latent HIV-1, MSCs must express HIV-1 entry markers, and show evidence of productive or latent HIV-1 infection. The effect of HIV-1 or HIV-1 proteins on MSC properties may also be indicative of HIV-1 infection. 
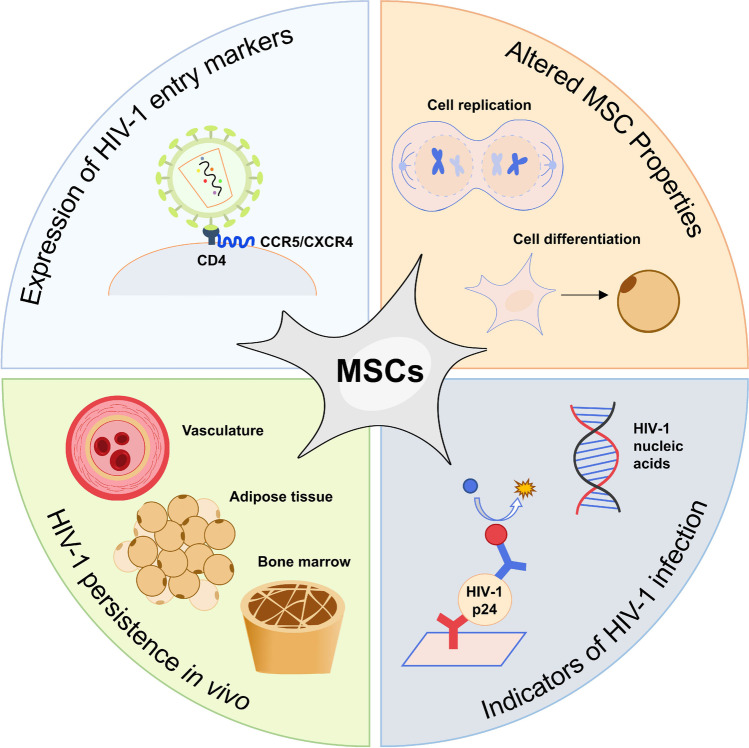

## Introduction

**Fig. 1:**
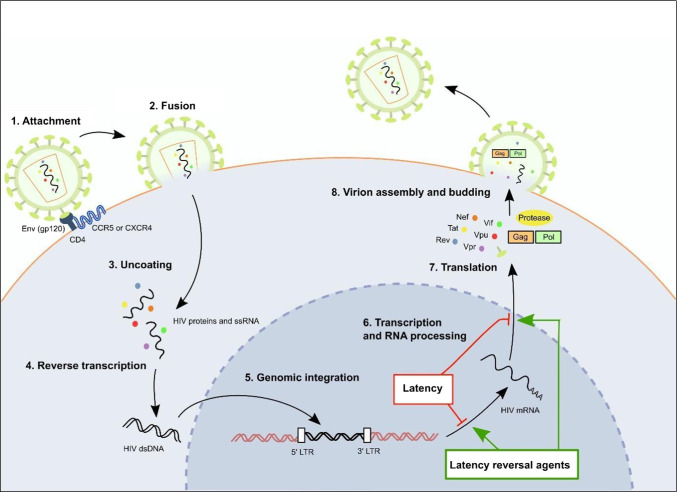
HIV-1 life cycle and latency. To enter host cells, the HIV-1 gp120 Env protein binds to the host CD4 receptor and CCR5/CXCR4 co-receptors (1). Once bound, the HIV-1 viral envelope fuses with the host membrane and uncoats, releasing HIV-1 proteins and ssRNA into the host cell cytoplasm (2-3). HIV-1 ssRNA is reverse-transcribed into the dsDNA proviral genome using HIV-1 reverse transcriptase (4) and can integrate into the dsDNA host genome in the nucleus (5). Host genomic DNA containing the integrated HIV-1 transcript can then undergo transcription, RNA processing and translation to produce viral proteins, which assemble with viral ssRNA to form mature, infectious HIV-1 virions (6-8). Alternatively, interference with gene expression through mechanisms such as epigenetic silencing and/or post-translational modifications, can cause the replication-competent provirus to remain latent within the host cell. Certain chemical compounds, termed latency reversal agents, can reverse silencing mechanisms to reactivate latent HIV-1. Abbreviations: CCR5: C-C-motif chemokine receptor type 5; CD4: cluster of differentiation type 4; CXCR4: C-X-C-motif chemokine receptor type 4; dsDNA: double stranded DNA; Env: HIV-1 envelope protein; Gag: HIV-1 group specific antigen; gp120: HIV-1 glycoprotein 120; LTR: long terminal repeat; mRNA: messenger ribonucleic acid; Nef: HIV-1 negative factor protein; Pol: HIV-1 polymerase; Rev: HIV-1 response element protein; ssRNA: single-stranded RNA; Tat: HIV-1 transactivating protein; Vif: HIV-1 viral infectivity factor protein; Vpr: HIV-1 viral protein R; Vpu: HIV-1 viral protein U

Human immunodeficiency virus (HIV)-1 primarily targets and infects cluster of differentiation (CD)4^+^ T cells, leading to compromised immune function and leaving the host vulnerable to opportunistic infections. Without treatment, immune function is gradually reduced and the individual advances to the final and most severe stage of the disease, acquired immune deficiency syndrome (AIDS) [1]. HIV-1 uses the machinery of CD4^+^ T cells to multiply and spread through the body. Entry of HIV-1 into CD4^+^ T cells occurs through binding of the HIV-1 envelope (Env) protein, gp120, to CD4, the primary cell surface receptor on the host cell (Fig. 1) [2, 3]. Subsequent co-receptor binding to C-C-motif chemokine receptor type 5 (CCR5) for R5-tropic viruses, to C-X-C-motif chemokine receptor type 4 (CXCR4) for X4-tropic viruses or to both co-receptors for R5X4 dual tropic viruses, results in fusion of the viral and CD4^+^ T cell membranes allowing transfer of the viral genome into the host cell [4, 5]. Inside the host cell, HIV-1 ribonucleic acid (RNA) is reverse transcribed into HIV-1 proviral deoxyribonucleic acid (DNA). Proviral DNA enters the host cell nucleus where it is integrated into the host cell genome. Upon integration, host RNA polymerase II enzymes transcribe the proviral DNA to form messenger RNA (mRNA). Viral mRNA is then translated by host cell translation machinery to form viral proteins. Viral proteins and transcribed viral single-stranded RNA (ssRNA) molecules move to the cell surface where they assemble to form immature, non-infectious virions. These virions bud from the host cell plasma membrane and undergo a protease-mediated maturation process to form mature, infectious virions that contain fully functional reverse transcriptase and integrase enzymes [6–8]. Refer to Fig. 1 for a detailed depiction of the HIV-1 life cycle.

HIV-1 persists in cells where it can adopt two different functional states, namely productive/replicative and latent [9]. The introduction of antiretroviral therapy (ART) and highly active antiretroviral therapy (HAART) has transformed HIV-1 into a chronic, well-managed disease by effectively reducing viral load and the effects of HIV-1 on the immune system. ART or HAART primarily target the productive/replicative state by reducing and/or inhibiting HIV-1 replication. However, these therapies do not eliminate all infected cells from the body despite suppressing viral load. To achieve effective viral suppression, a treatment adherence rate of 95% is recommended [10, 11]. Viral rebound is largely caused by HIV-1 latency, which is defined as a non-productive, reversible state of HIV-1 infection characterised by no or low-level expression of viral proteins and a lack of viral replication. Some studies have also expanded the definition of latent HIV-1 to include transcription- and translation-competent proviruses, which are not fully replication-competent, but produce viral mRNA and proteins [12, 13]. Long term persistence of HIV-1 is supported by infected cellular compartments harbouring latent HIV-1, termed cellular reservoirs [14, 15].

Due to a multitude of factors, including epigenetic regulation and/or silencing, HIV-1 reservoirs exhibit low-level or zero HIV-1 gene expression in patients receiving optimal ART [16, 17]. However, gene expression and viral replication resume following treatment interruption; thus, HIV-1 reservoirs constitute a challenge to the complete eradication of the virus from infected patients. Latency reversal agents (LRA) can be used to reactivate gene expression of latent HIV-1, rendering the virus vulnerable to elimination by the host immune system and ART, an approach labelled “shock and kill”. Additionally, LRAs can be used to distinguish between latently infected cells and cells either uninfected with HIV-1 or refractory to HIV-1 infection [18]. Complete eradication of the virus is difficult, since mechanisms contributing to HIV-1 latency are not fully understood and LRAs must effectively eliminate HIV-1 from a variety of cell types and tissues to be effective [19]. Figure 1 outlines the effect that latency and LRAs have on HIV-1 gene expression during the HIV life cycle.

A thorough understanding of the HIV-1 reservoir will facilitate the development of new strategies to detect, reduce and eliminate the reservoir, ultimately leading to HIV-1 curative therapies. Although immune cells derived from lymphoid and myeloid progenitors have been thoroughly studied as HIV-1 reservoirs, few studies have examined whether mesenchymal stromal/stem cells (MSCs) can assume this function [20]. MSCs are a heterogeneous population of cells containing a subset of non-hematopoietic multipotent adult stem cells. Although originally isolated from bone marrow, MSCs have been found to reside in virtually all post-natal human tissues and organs [21, 22]. MSCs are easily attainable, can be isolated from various tissues, and show promise therapeutically due to their differentiation capacity and ability to activate endogenous progenitor cells through paracrine signalling [23]. Furthermore, the ability of MSCs to migrate to sites of inflammation throughout the body, including the central nervous system (CNS), could facilitate viral dissemination if MSCs are indeed an additional HIV-1 reservoir [22, 24].

Many individuals infected with HIV-1 while receiving HAART still manifest clinical features such as reduced bone mineral density, altered lipid metabolism (lipodystrophy, dyslipidemia) and haematological abnormalities [25–27]. This was initially thought to be the result of ART toxicity; however, their occurrence in ART naïve HIV-infected people suggests that the virus plays a role in driving these abnormalities [28, 29]. Adipose tissue has been suggested to be a reservoir for HIV-1 [30, 31]. Adipocytes express HIV-1 entry receptors and co-receptors [32] and can be infected *in vitro* with HIV-1 [33], but the presence of HIV-1 in these cells has not been reported [34, 35]. Adipose tissue is not only involved in metabolic homeostasis but also contains MSCs that support bone and lipid homeostasis and that can differentiate into osteoblasts and adipocytes [36, 37]. Infection of MSCs in the bone marrow or adipose tissue niche by HIV-1 could explain these comorbidities. To examine whether MSCs contribute to the HIV-1 reservoir, this review evaluates studies addressing HIV-1 receptor and co-receptor expression on MSCs, and whether these cells can become infected with and harbour the virus.

## HIV-1 Reservoirs

### Lymphoid Progenitors

CD4^+^ T cells, which arise from lymphoid progenitors, represent the largest and best characterised HIV-1 reservoir. Resting CD4^+^ T memory cells were initially regarded as the main T cell population harbouring latent HIV-1 [38–41]. However, viral latency has also been observed in activated and proliferating [42, 43] as well as in regulatory CD4^+^ T cells [44, 45]. When infected T cells undergo clonal expansion, the integrated proviral genome is transferred to daughter cells [46–49]. In lymph nodes, follicular helper T cells have been shown to contribute to the viral reservoir [50, 51]. CD4^+^ resident memory T cells from ART-treated women whose plasma viral load was undetectable for up to 10 years, have been identified in cervical tissues; this may constitute another cellular reservoir [52]. Considering the wide distribution of resident CD4^+^ memory T cells in tissues throughout the body, this cell subset may contribute to persisting HIV-1 reservoirs [52, 53]. Notably, CD4^+^ T cells undergoing an effector-to-memory cell type transition have temporarily upregulated CCR5 expression and downregulated global gene expression, making them ideal for harbouring latent HIV-1 [54].

### Myeloid Progenitors

Cells derived from myeloid progenitors, namely, macrophages, monocytes, basophils, and mast cells, can express HIV-1 receptors and co-receptors [55–60]. Despite this observation, the contribution of these cells to the HIV-1 reservoir is not fully understood.

Although macrophages exhibit lower CD4 expression than CD4^+^ T cells, macrophage tropic viruses can adapt to this low level of expression to infect macrophages [61, 62]. The self-renewal capabilities of tissue-resident macrophage populations makes them ideal for harbouring latent HIV-1 [63, 64]. Minor populations of HIV-1 isolated from patients undergoing ART interruption are macrophage-tropic and express macrophage markers indicative of macrophage origin [65]. For instance, a study using penile tissue from ART-suppressed individuals recovered replication-competent HIV-1 from urethral macrophages [66]. This suggests that macrophages can form a latent HIV-1 reservoir in patients undergoing ART, with re-activation of the virus following treatment interruption, although further studies are needed to confirm this.

Classical monocytes (CD14^+^ CD16^−^), which comprise the majority of the circulating monocyte pool, are derived from the bone marrow and circulate in the bloodstream before migrating into surrounding tissues [67]. Migration of classical monocytes to the tissues is initiated during both steady-state and inflammatory conditions to produce dendritic cells or macrophage progeny [67, 68]. Despite being permissive to HIV-1 infection, circulating classical monocytes do not represent a stable reservoir since they have a short half-life and undergo apoptosis after approximately 24 hrs if migration into surrounding tissues has not taken place [67, 69–71]. Although infected classical monocytes do not technically represent an HIV-1 reservoir, they can cross the blood-brain barrier and differentiate into macrophages capable of transferring HIV-1 to astrocytes, microglia, and dendritic cells within the CNS, which form a prominent HIV-1 reservoir due to their longevity [72–77].

The role of other myeloid progenitor-derived cells such as basophils and mast cells in HIV-1 persistence is unclear. Some studies have shown that basophils and mast cells can express HIV-1 receptors and co-receptors on the cell surface [58–60]. Additionally, mast cells express cell surface HIV-1 attachment factors, such as heparan sulfate proteoglycan (HSPG) and α4β7 integrin [78]. Expression of HIV-1 receptors/co-receptors and/or attachment factors suggests that basophils and mast cells are susceptible to HIV-1 and may play a supporting role in the formation of HIV-1 reservoirs by disseminating the virus and trans-infecting susceptible CD4^+^ T cells [58–60, 78].

### Hematopoietic Stem/Progenitor Cells

The self-renewal capabilities of haematopoietic stem/progenitor cells (HSPCs) make them ideal HIV-1 reservoir candidates. The jury is still out as to whether HSPCs can become infected with HIV-1 and function as an HIV-1 reservoir [79]. Some studies claim that HSPCs can become infected with HIV and contribute to an HIV-1 reservoir [80, 81], whereas others were unable to find evidence of HIV infection in HSPCs [82, 83]. However, research is starting to identify specific HSPC populations that express sufficient levels of CD4, CCR5 and CXCR4 to become infected with HIV-1 [84–88]. A relatively recent study that screened for integrated HIV-1 provirus found the presence of productive and latent infection in freshly isolated HSPCs from HIV-1-infected donors receiving ART. They reported that specific HSPC populations can be infected with and produce HIV-1 and harbour latent proviral genomes [84]. The role of HSPCs in HIV persistence and the identification of HSPC subpopulations that serve as HIV reservoirs needs to be clarified before HSPCs can be regarded as long-lived viral reservoirs. In addition, HSPCs infected with and/or harbouring latent HIV-1 may play a role in haematological co-morbidities observed in HIV-1 infected individuals; however, these co-morbidities could also result from the indirect effect of HIV-1 on other cell types [25, 81, 89, 90].

## Techniques Used to Detect HIV-1 Reservoirs

### Detecting Viral RNA/DNA and Genomic Integration

Polymerase chain reaction (PCR) techniques such as reverse transcription PCR (RT-PCR), quantitative PCR (qPCR), reverse transcription, quantitative PCR (RT-qPCR) and digital droplet PCR (ddPCR) are commonly used to detect the presence of HIV-1 RNA or DNA in host cells upon infection [64, 91]. These techniques can also be used to test for the integration of HIV-1 DNA into the human genome. RT-PCR and RT-qPCR measure levels of viral RNA in a non-quantitative and quantitative manner, respectively, before reverse-transcription to proviral DNA, while quantitative PCR (qPCR) and ddPCR measure levels of viral DNA either before or after integration into the host genome [91]. These techniques are cost-effective but can overestimate viral titre, since not all HIV-1 proviruses are replication-competent [64]. Additionally, the above-mentioned techniques are often not specific for integrated HIV-1 [64]. Nested *Alu-gag* PCR, a technique which combines PCR detection of HIV-1 *gag* gene sequences with detection of repetitive *Alu* sequences in the human host, can be employed to quantify integrated DNA to provide a more accurate estimation [92]. Additionally, to decrease the overestimation that often occurs with conventional PCR techniques, a newly introduced Q4PCR technique targets four conserved regions of the HIV-1 genome in an approach that combines both qPCR and next-generation sequencing (NGS) [43, 93].

### Detecting Productive Infection

Conventional and digital p24 enzyme-linked immunosorbent assay (ELISA) methods are used to detect productive HIV-1 infection by quantitatively measuring levels of the HIV-1 capsid protein (p24) in plasma, serum, or cell culture supernatant [94]. An estimation of the productive viral load can be calculated from p24 levels; however, defective proviruses can still produce p24 proteins, leading to an overestimation of infectious viral levels [95]. A recent technique, the *Tat/rev* induced limiting dilution assay (TILDA), has been introduced to avoid overestimation of the productive viral titre. TILDA reduces productive viral titre overestimation by measuring the levels of viral *Tat/rev* multiply-spliced RNA (msRNA) [96]. *Tat/rev* msRNA is transcribed in productively but not latently infected cells unless these cells are activated with LRAs. The TILDA assay utilises this principle to measure the frequency of productively infected cells [96]. TILDA is accurate and sensitive; however, viral reservoir amounts can still be overestimated due to the occurrence of defective transcripts [70].

### Detecting Latent Infection

To measure the size of the latent reservoir, the TILDA assay can be used on cells that have been exposed to LRAs such as phytohemagglutinin (PHA) which reactivates gene expression of latent HIV-1 [70]. The TILDA assay can become a powerful tool for determining the percentage of productively and latently infected cells when combined with LRAs [70]. Full-length individual proviral sequencing (FLIPS), an alternative for estimating the viral load of latent reservoirs, utilises NGS to detect near full-length proviral sequences [97]. FLIPS determines the proportion of replication-competent virus by identifying if there are defects within integrated HIV-1 proviral sequences that would potentially render the virus incapable of replication [97]. Using this principle, FLIPS can be used to detect replication-competent latent virus lying dormant within cells. This technique is time-consuming but gives highly accurate results; however, errors may occur during NGS library construction [43, 64].

HIV-1 quantitative viral outgrowth assays (qVOAs) in conjunction with p24 ELISA are considered to be the gold standard for measuring replication-competent latent HIV-1 [43]. Initial steps involve activation of infected cells with a mitogen and co-culturing these cells in serial dilution with PHA-activated, uninfected peripheral blood mononuclear cells (PBMCs) [98]. These steps induce reactivation and expansion of latent HIV-1, which can then be quantified using p24 ELISA techniques which provides an estimated size of the viral reservoir [99]. Table 1 summarises the different techniques along with relevant studies used to detect HIV-1 integration, infection, and latency in CD4^+^ T cells and where available, in MSCs.

**Table 1:** Studies detecting HIV-1 infection and latency in CD4^+^ T cells and MSCs

Technique	Purpose	Target	Technique used on activated cells*?	Relevant studies using CD4^+^ T cells to establish techniques	Relevant studies using MSCs (Papers discussed in this review)
PCR	Presence of viral DNA in cells exposed to HIV-1	HIV-1 specific DNA such as *gag*	No		BM-MSCs: [100] Vessel wall-derived MSCs: [101] Adipose cells/preadipocytes: [32] BM-derived stromal cells: [102, 103]
qPCR	Genomic integration of HIV-1 into the host genome	Conserved genomic regions of HIV-1 DNA, including *pol*, *gag*, *tat* and/or episomal 2-LTR circles	No	[104–110]	ASCs: [111]
ddPCR		Conserved genomic regions of HIV-1 DNA, including *pol* and *gag*, and/or episomal 2-LTR circles	No	[112–114]	
Nested qPCR		*Alu* elements in the human genome and HIV-1 *gag* and/or LTR DNA regions	No	[92, 115, 116]	Vessel wall-derived MSCs: [101] BM-MSCs: [117]
Q4PCR		HIV-1 packaging signal, *gag*, *pol*, and *env* DNA regions	No	[93]	
ELISA (conventional and digital)	Productive infection	p24	No	[118–121]	BM-MSCs: [117, 122] Vessel wall-derived MSCs: [101] ASCs: [111] Adipose cells/preadipocytes: [32] BM-derived stromal cells: [102, 103, 123]
TILDA		*Tat* and *rev* multiply spliced RNA	Yes or no	[96]	
	Latent infection				
FLIPS		Full-length HIV-1 genomic DNA	No	[124]	
qVOA and ELISA		p24	Yes	[125–128]	

*Activated with latency reversal agents, such as PHA and PMA

Abbreviations: *ASCs* Adipose-derived mesenchymal stromal/stem cells, *(BM)-MSCs* (Bone marrow-derived) mesenchymal stromal/stem cells, *ddPCR* digital droplet polymerase chain reaction, *DNA* deoxyribonucleic acid, *ELISA* enzyme-linked immunosorbent assay, *env* HIV-1 envelope protein, *FLIPS* full-length individual proviral sequencing, *gag* HIV-1 group specific antigen, *LTR* long terminal repeat, *MSCs* mesenchymal stromal/stem cells, *PHA* phytohaemagglutinin A, *PMA* phorbol 12-myristate 13-acetate, *pol* HIV-1 polymerase, *PCR* polymerase chain reaction, *qPCR* quantitative PCR, *Q4PCR* combined quadruplex qPCR, *qVOA* quantitative viral outgrowth assay, *rev* HIV-1 response element protein, *RNA* ribonucleic acid, *tat* HIV-1 transactivating protein, *TILDA* *Tat/rev* induced limiting dilution assay

## Mesenchymal Stromal/Stem Cells

Adult stem cells maintain tissue homeostasis by affecting tissue specific turnover and repair. The fate of stem cells is controlled by their specialised microenvironment, referred to as the stem cell niche, through cell-to-cell interactions and molecular signalling [129]. Increasing interest in MSCs has led to inconsistencies regarding their nomenclature. To clarify the terminology, the International Society for Cellular Therapy (ISCT) proposed that fibroblast-like plastic adherent cells, regardless of tissue origin, be named multipotent mesenchymal stromal cells, while reserving the term mesenchymal stem cell for a subset of cells that clearly demonstrate stem cell properties [130, 131].

### Sources of MSCs

MSCs are commonly isolated from the bone marrow [132] or adipose tissue [133, 134]; however, MSCs can also be isolated from neonatal-derived tissues such as umbilical cord blood (UCB) [135] and Wharton’s Jelly [136, 137], and from vessel walls [138, 139]. The International Federation for Adipose Therapeutics and Science (IFATS) has proposed the use of the term “adipose-derived (mesenchymal) stromal cells” (ASCs) to identify the plastic-adherent, multipotent cell population isolated from adipose tissue [140]. MSCs isolated from bone marrow are referred to as bone marrow-derived MSCs (BM-MSCs).

### *In Vitro* Characterisation of MSCs

Stromal cells are classified as MSCs *in vitro* if they meet the following minimal criteria proposed by the ISCT: they need to 1) be plastic adherent in culture; 2) display a specific immunophenotype, with ≥95% of the MSC population being positive for CD105, CD73 and CD90 and ≤2% expressing CD45, CD34, CD14 or CD11b, CD79α or CD19 and HLA-DR; and 3) show multilineage differentiation capacity into adipocytes, osteoblasts and chondroblasts [141]. In 2013, IFATS and ISCT made a joint statement to define stromal cells specifically from adipose tissue [140]. They proposed the use of multi-colour flow cytometry analysis to confirm the phenotype of ASCs along with qualitative and quantitative evaluation of differentiation capacity. They defined ASCs as being positive for the cell surface markers CD90, CD73, CD29, CD105/CD13 and CD44 and negative for CD45 and CD31. Furthermore, they suggested that by including CD36 and CD106, MSCs from bone marrow and adipose tissue could be distinguished from one another: BM-MSCs are positive for CD106 and negative for CD36, whereas ASCs are negative for CD106 and positive for CD36.

### *In Vivo* Characterisation of MSCs

The *in vivo* localisation, identification, and role of MSCs in their multiple anatomical locations is still poorly characterised. Whether they constitute a specific homogenous cell type, or a heterogeneous population, still needs to be elucidated. In search of the *in vivo* identity of MSCs, it has been postulated that MSCs exist throughout the body as pericytes due to their perivascular location [142, 143]. In the bone marrow niche, MSCs support haematopoiesis through the secretion of cytokines, growth factors and chemokines, and participate in bone remodelling and metabolism [144–146]. In the adipose niche, ASCs reside in the stromal vascular fraction (SVF) as multipotent precursor cells involved in adipocyte turnover [147, 148].

## MSCs Express Receptors Required for HIV-1 Entry *In Vitro*

Different techniques can be used to detect, quantify, and characterise cell surface receptors [149]. Cell surface receptors are proteins, and thus they are synthesised via the same process as other proteins; DNA coding for the receptor is transcribed to mRNA, followed by translation of mRNA to the protein product. The detection of receptors can be performed at the mRNA level and at the protein level. RT-PCR/RT-qPCR detects mRNA levels as an indication of receptor transcription; however, the amount of mRNA does not necessarily correlate with protein production due to translational control, amongst other mechanisms [150, 151]. Protein expression of cell-surface or internalised receptors can be evaluated using immunostaining techniques or binding assays. Immunostaining techniques detect increased receptor-protein levels on sections of biological tissues using immunohistochemistry (IHC) or on intact cells using immunocytochemistry (ICC), whereas binding assays, such as flow cytometry, detect the presence of cell-surface receptors on live cells. These techniques use labelled (e.g., with a fluorescent dye) antibodies that specifically recognise the receptor.

It is important to understand whether MSCs express the CD4 receptor and CCR5/CXCR4 co-receptors required for HIV-1 entry before evaluating whether they can harbour latent HIV-1. Entry of HIV-1 into a host cell requires binding of HIV-1 to the CD4 receptor followed by CCR5 and/or CXCR4 co-receptor binding. Early during HIV-1 infection, CCR5 is predominantly utilised for HIV-1 entry. As infection progresses, co-receptor tropism changes can occur, increasing the frequency of CXCR4 utilisation [152]. Expression of CD4, CXCR4 and CCR5 mRNA by MSCs has been observed *in vitro* [101, 102, 111, 117, 153–161]. However, the reported level of expression and localisation of these receptors/co-receptors is inconsistent.

### Expression of CD4

Using RT-qPCR, Cotter *et al.* found that HIV-1-exposed and unexposed human BM-MSCs express CD4 at the mRNA level [117]. CD4 glycoproteins were also detectable on the cell surface using ICC. In contrast, studies by Scadden *et al.* and Gibellini *et al.* failed to detect CD4 on the surface of human BM-MSCs and vessel wall-derived MSCs by flow cytometry [101, 102]. However, when the cells were fixed and permeabilised, intracellular CD4 was detected by flow cytometry in 20% of MSCs [101]. Additionally, CD4 mRNA was detectable in these MSCs using PCR and RT-qPCR techniques. The authors concluded that while CD4 was mainly expressed intracellularly, the very low levels expressed extracellularly may have been below the detection limit of flow cytometry [101]. Based on the above-mentioned findings, human MSCs appear to predominantly express CD4 intracellularly; however, additional studies are needed to address discrepancies in cell surface CD4 detection when using different experimental techniques, such as flow cytometry and ICC.

### Expression of CCR5

Contradictory results have been reported regarding CCR5 expression on human BM-MSCs. Sordi *et al*. and Karnoub *et al*. failed to detect CCR5 mRNA and protein on human BM-MSCs [156, 162]; whereas other studies suggest that BM-MSCs are capable of both CCR5 mRNA and protein expression, albeit at varying levels between studies [117, 160, 163, 164]. Similar inconsistencies in CCR5 expression have been observed in murine BM-MSCs. Some studies reported low or negligible expression of cell surface CCR5 on murine BM-MSCs by flow cytometry [164, 165], while Alexeev *et al*. reported detectable expression [159]. A thorough study on murine BM-MSCs demonstrated expression of CCR5 mRNA and localisation of the CCR5 co-receptor on the cell surface and in the cytoplasm using a combination of RT-PCR, flow cytometry and ICC, respectively [166]. Further insight into the CCR5 expression profiles of human BM-MSCs is needed and should use a similar approach to that of Ji *et al*., whereby complementary techniques are used to verify CCR5 1) mRNA expression, 2) protein expression and 3) localisation of the co-receptor within the cell [166].

A paucity of research exists on CCR5 expression profiles of MSCs and MSC-like cells isolated from sources other than bone marrow, namely dermal MSCs, vessel wall-derived MSCs and ASCs. A single pilot study on human dermal MSCs found by flow cytometry that the cells had negligible CCR5 cell surface expression [167]. On the other hand, RT-qPCR and flow cytometry analysis of human vessel wall-derived MSCs indicated that these cells were capable of CCR5 mRNA and cell surface protein expression [101]. Hazan *et al*., Maurin *et al*., and Munier *et al*. studied the expression profiles of preadipocytes [32–34]. The methods used to isolate these preadipocytes were similar to those used to isolate ASCs [133, 134]. Thus, the cells they classified as preadipocytes may have contained a subset of ASCs. Maurin *et al*. demonstrated the presence of CCR5 mRNA by RT-PCR and cell surface CCR5 by ICC, while Hazan *et al*. and Munier *et al*. found low or undetectable levels of CCR5. A study by Kroeze *et al*. using ASCs characterised according to ISCT guidelines, demonstrated negligible expression of cell surface CCR5 [167]. Additional studies are needed to confirm whether characterised ASCs also express CCR5 mRNA and whether co-receptor expression can be detected intracellularly in addition to being located on the cell surface.

### Expression of CXCR4

According to several studies, 26-96% of human BM-MSCs express CXCR4 on the cell surface [153, 154, 156, 164, 168, 169]. In contrast, Wynn *et al.* and *Shi et al*. found respectively that 83-98% and 95.9% of human BM-MSCs expressed CXCR4 intracellularly, with less than 1% of cells displaying extracellular expression [155, 169]. Inconsistencies in results may be due to different experimental techniques and culturing conditions, since extracellular CXCR4 has been shown to decrease with passage number in MSCs [153, 159, 170]. Depletion of cytokines, chemotactic and growth factors during cell culture has been proposed as a possible reason for the loss of extracellular CXCR4 [169]. The interaction of CXCR4 with cyclic AMP, prostaglandin E2 and nitric oxide mediators, interleukin (IL)-3, IL-6, IL-7, and stem cell factor (SCF) cytokines, and growth factors such as hepatocyte growth factor (HGF), commonly occur *in vivo*, inducing expression and cell surface localisation of the co-receptor [170–173].

Interactions of CXCR4 with its ligand, stromal-derived factor 1 (SDF-1), additionally play a large, dynamic role in the expression and cellular localisation of CXCR4. The SDF-1-CXCR4 axis is crucial for MSC chemokine-mediated migration to sites of injury [154, 155, 170, 174]. SDF-1-induced chemokine signals increase expression of CXCR4 on the surface of murine MSCs [158]. Upon binding SDF-1, MSC CXCR4 expression is decreased, and the co-receptor is internalised through endocytosis or micropinocytosis [157, 175]. CXCR4 is then internally degraded by lysosomes or is recycled back to the cell surface [175, 176].

### Effect of HIV-1 on Expression Of Entry Receptors

Whether exposure to HIV-1 or HIV-1 proteins can enhance expression and cell surface localisation of HIV-1 receptors/co-receptors on MSCs is an area of active research. The interaction of HIV-1 gp120 with CXCR4 is of particular interest, since SDF-1 and gp120 both bind to CXCR4, albeit at non-intersecting N-terminal regions [172, 177–181]. An early study by Tarasova *et al.* found that gp120, like SDF-1, has a high affinity for CXCR4 and can cause irreversible co-receptor internalisation [182]. Other studies further discovered that the internalisation of CXCR4, as the result of gp120 binding, reduced SDF-1-CXCR4 interactions and migration of B cells, CD4^+^ T cells and monocytes [183–185]. However, this may not be the case with MSCs. Recently, Li *et al.* found that exposure of MSCs to 100 ng/mL X4- and R5-tropic HIV-1 gp120 glycoproteins increased external expression of CXCR4 by approximately 2-3-fold as measured by flow cytometry, and enhanced MSC migration [154]. This study suggested that exposure of MSCs to gp120 may prime HIV-1 to undergo a R5- to X4-tropism change by increasing the expression of CXCR4 on the MSC cell surface [154]. If this theory is valid, it would be advantageous for HIV-1 to not only increase cell surface expression/localisation of the CXCR4 co-receptor, but also the CD4 receptor, since HIV-1 entry is initiated by sequential binding to CD4 followed by CXCR4/CCR5. A study by Cotter *et al.* has shown that this is plausible, as MSCs exposed to high viral load (HVL; 100 000 to 150 000 copies/mL) HIV-1 sera for at least 72 hrs increased expression of extracellular CD4, as detected by ICC [117]. However, further studie*s* are required to assess the validity of this hypothesis and to unravel the effects of HIV-1 and HIV-1 proteins on MSC receptor/co-receptor expression and localisation, particularly *in vivo*. Table 2 outlines studies examining expression of the HIV-1 receptors/co-receptors by human MSCs, and includes the experimental techniques used for detection of cell surface and intracellular expression.

**Table 2:** HIV-1 receptor and co-receptor expression in cultured human MSCs

MSC source	Receptor/co- receptor	Technique		Outcome	Reference
		Intracellular	Extracellular		
BM-MSCs	CD4	RT-qPCR	Immunocytochemistry on HIV-1 exposed/unexposed cells	Detection of mRNA. Cell surface expression, which increased with HIV-1 exposure.	[117]
		PCR	Flow cytometry	Detection of mRNA. Failure to detect cell surface expression.	[102]
	CCR5		Immunocytochemistry	Low expression on cell surface.	[117]
		RT-PCR	Flow cytometry	Failure to detect mRNA and cell surface expression.	[156]
		RT-qPCR	Immunocytochemistry	Detection of mRNA. Cell surface expression.	[160]
			Immunocytochemistry	Failure to detect cell surface expression.	[162]
		Immunocytochemistry		High expression in cells.	[163]
			Flow cytometry	Cell surface expression on approximately 78% of cells.	[165]
	CXCR4	RT-PCR	Flow cytometry	Failure to detect mRNA. Cell surface expression on 1% of cells. Intracellular expression in 83-98% of cells.	[155]
		RT-PCR	Flow cytometry	Detection of mRNA. Cell surface expression on approximately 26% of cells.	[156]
		RT-PCR	Flow cytometry	Detection of mRNA. Cell surface expression on 43% of cells.	[153]
		RT-qPCR	Immunocytochemistry	Detection of mRNA. Cell surface expression.	[160]
			Immunocytochemistry	Cell surface expression.	[117]
		RT-qPCR	Flow cytometry on 100 ng/mL gp120 treated/untreated cells	Detection of mRNA. Cell surface expression on 84% of cells. Treatment with gp120 upregulated surface expression by 2-3-fold.	[154]
		RT-qPCR	Flow cytometry	Low levels of mRNA. Cell surface expression on approximately 2% of cells.	[186]
		RT-qPCR	Flow cytometry	Detection of mRNA. Cell surface expression on approximately 29.8% of cells.	[168]
		RT-qPCR and flow cytometry	Flow cytometry	Detection of mRNA. Intracellular expression in 95.9% of cells. Cell surface expression on 0.1% of cells.	[169]
			Flow cytometry	Cell surface expression on approximately 96% of cells.	[164]
Preadipocytes (may contain a subset of ASCs)	CD4	RT-PCR		Detection of mRNA.	[32]
		RT-PCR	Immunocytochemistry	Detection of mRNA. Failure to detect cell surface expression.	[33]
		RT-PCR		Failure to detect mRNA.	[34]
	CCR5	RT-PCR		Low levels of mRNA.	[32]
		RT-PCR	Immunocytochemistry	Detection of mRNA. Cell surface expression.	[33]
		RT-PCR		Failure to detect mRNA.	[34]
	CXCR4	RT-PCR		High levels of mRNA.	[32]
		RT-PCR	Immunocytochemistry	Detection of mRNA. Cell surface expression.	[33]
		RT-PCR		Detection of mRNA.	[34]
ASCs	CCR5		Flow cytometry	Negligible cell surface expression.	[167]
	CXCR4		Flow cytometry	Cell surface expression on approximately 1.21% of cells.	[167]
Dermal MSCs	CCR5		Flow cytometry	Negligible cell surface expression.	[167]
	CXCR4		Flow cytometry	Cell surface expression on approximately 1.31% of cells.	[167]
Vessel wall-derived MSCs	CD4	RT-qPCR and flow cytometry	Flow cytometry	Detection of mRNA. Intracellular expression in 20% of cells. Failure to detect cell surface expression.	[101]
	CCR5	RT-qPCR	Flow cytometry	Detection of mRNA. High levels of expression on cell surface.	
	CXCR4	RT-qPCR	Flow cytometry	Detection of mRNA. High levels of expression on cell surface.	
Fetal blood MSCs	CXCR4	RT-PCR and flow cytometry	Flow cytometry	Detection of mRNA. Cell surface expression on 23-25% of cells. Intracellular expression in 78-83% of cells.	[158]
Fetal BM-MSCs	CXCR4	RT-qPCR and flow cytometry	Flow cytometry	Low levels of mRNA. Cell surface expression on 3.8% of cells. Intracellular expression in 50-90% of cells.	[157]

Abbreviations: *ASCs* adipose-derived mesenchymal stromal/stem cells, *(BM)-MSCs* (Bone marrow-derived) mesenchymal stromal/stem cells, *CCR5* C-C-motif chemokine receptor type 5, *CD4* cluster of differentiation type 4, *CXCR4* C-X-C-motif chemokine receptor type 4, *gp120* HIV-1 glycoprotein 120, *mRNA* messenger ribonucleic acid, *MSCs* mesenchymal stromal/stem cells, *PCR* polymerase chain reaction, *RT-PCR* reverse transcription PCR, *RT-qPCR* reverse transcription quantitative PCR, *qPCR* quantitative PCR

## *In Vitro* Infection of MSCs with HIV-1

Since MSCs express CD4, CXCR4 and CCR5 receptors/co-receptors that are required for HIV-1 infection [117], studies *in vitro* have sought to establish whether MSCs could be infected with the virus. A limited number of studies can be found in the literature investigating the infectability of MSCs by HIV-1. Early studies focused on BM-derived stromal cells and the effect HIV-1 had on these cells. Recent studies have focused more on the effect of HIV-1 and HIV-1 proteins on MSC characteristics which will be described in the subsequent section.

### Early Experiments

In the early 1990’s, Scadden *et al.* reported that primary human BM-derived stromal cells are susceptible to HIV infection [102]. Since these cells were characterised as being free of monocyte/macrophage or endothelial cell contamination, they were referred to as BM stromal fibroblasts. MSCs are morphologically indistinguishable from fibroblasts and share surface immunophenotype, proliferation and differentiation capacity. It has thus been proposed that MSCs may originate from a fibroblast lineage [187]. The BM stromal fibroblasts isolated and investigated by Scadden *et al.* may be representative of the cells we label as BM-MSCs today. Cultures of primary BM stromal fibroblasts were positive for p24 antigen when exposed to X4-tropic HIV-1_IIIB_. When co-cultured with either H9 lymphoid or KG-1 myeloid cells, the BM stromal fibroblasts were capable of transferring HIV to lymphoid or myeloid cells. Using PCR, HIV-1 DNA was detectable for up to 4 weeks post-infection. However, following exposure to R5-tropic HIV-1_Ba-L_ or HIV-1_RJ9533_ strains, the authors could not detect p24 antigen or viral DNA by PCR, indicating that exposure to HIV-1 failed to result in HIV-1 infection.

Canque *et al.* investigated whether primary human BM-derived stromal cells (containing a mixture of stromal cells, fibroblasts, endothelial cells, and macrophages) are susceptible to HIV [103]. Once the BM-derived stromal cultures reached confluency, non-adherent cells were removed and the cultures exposed overnight to either R5-tropic HIV-1_Ada_, HIV-1_Ba-L_ and HIV-1_JR-FL_ strains or X4-tropic HIV-1_LAI_ and HIV-1_MN_ strains. BM-derived stromal cells were positive for p24 antigen when exposed to R5-tropic HIV-1 strains but failed to produce detectable p24 when exposed to X4 tropic HIV-1 stains. To identify which subset of BM-derived stromal cells was producing virus, ICC was used and identified macrophages (CD14^+^CD68^+^CD45^+^TE7^-^) as being responsible for the detected p24 expression (Kal-1^+^) whereas fibroblasts (CD14^-^CD68^lo^CD45^-^TE7^-^) were p24-negative (Kal-1^-^). In contrast to Scadden *et al.* who reported that stromal fibroblasts supported HIV-1_IIIB_ infection and replication, Canque *et al.* showed that BM-derived stromal cells depleted of macrophages did not produce p24 when exposed to X4-tropic HIV-1_LAI_ or R5-tropic HIV-1_Ba-L_. However viral DNA (123 bp, HIV-1 *pol*) could be detected and coculture with PHA-treated lymphocytes could rescue viral levels only in HIV-1_Ba-L_ exposed macrophage depleted BM-derived stromal cells. Exposure to higher HIV-1_LAI_ virus titres resulted in low levels of p24 which could mean that there is a correlation between infectability and virus titre. Marandin *et al.* confirmed the studies by Canque *et al.* showing that productive infection could be established in BM-derived stromal cells by p24 expression when exposed to R5-tropic but not X4-tropic HIV-1 strains [123].

Experiments by Bahner *et al.* demonstrated that incubation of BM-derived stromal cell monolayers with R5-tropic HIV-1_JR-FL_ at an MOI of 1 could initiate a productive low level viral infection which could not be achieved at lower MOIs (MOI < 1) [122]. The low-level infection was detectable by p24 antigen ELISA 3 weeks after inoculation and could be amplified by co-culturing the BM-derived stromal cells with monocytes, suggesting that the BM-derived stromal cells were infected with HIV-1 [122]. In contrast, co-culturing with stromal cells incapable of supporting HIV-1 infection, such as murine stromal cells, did not rescue viral levels [122]. These experiments show that BM-derived stromal cells can be infected *in vitro* with HIV-1 at high MOI, but that the level of virus being produced is low. Low level expression of viral proteins and viral replication could be an indicator of viral persistence through latency [188]; however, identification of latency was not the focus of this paper and was not evaluated further.

Research by Wang *et al.* supported the findings of Bahner *et al.* [100]. The authors exposed BM-derived stromal cells to dual-tropic HIV-1_RF_ (MOI = 1) 24 hrs post-culturing for a week. The viral levels were approximated post-exposure by quantifying p24 on a weekly basis for 2-3 weeks using ELISA. To amplify HIV-1, 2- to 3-week post-exposure BM-derived stromal cells were co-cultured with uninfected T cells and p24 levels were measured using ELISA after a week of co-culturing. Negligible or low levels of p24 were detectable in the BM-derived stromal cells without co-culture, while stromal cells co-cultured with T cells demonstrated amplification of viral levels for up to 3-4 weeks post-infection. PCR was performed to test for the presence of HIV-1 proviral DNA within the BM-derived stromal cells. HIV-1 DNA products of 115 bp were detected. These results collectively suggested viral persistence in the BM-derived stromal cells and transmission to T cells. To test which BM stromal subpopulations harboured HIV-1 DNA, the authors performed *in situ* hybridisation using an HIV-1 DNA fragment as a probe. Stromal subpopulations which tested positive for HIV-1 DNA also stained positive for CD83, a dendritic cell/dendritic precursor marker detected using immunostaining methods. Thus, the authors concluded that CD83+ subpopulations may be the cell type in BM-derived stromal cells responsible for harbouring HIV-1.

The early studies by Scadden, Canque, Bahner, Wang and colleagues, which utilised adherent BM-derived stromal cells for experiments, seemed to indicate that BM-MSCs could be infected with HIV-1 [100, 102, 103, 122]. However, the studies did not pinpoint which BM-derived stromal cells were infected by confirming the immunophenotype of the infected cells, as this was not considered necessary at the time. Stroma isolated from bone marrow may contain fibroblasts, macrophages, adipocytes, and endothelial cells, in addition to MSCs. These cells can have overlapping characteristics. For instance, CD83+ stromal cell fractions can contain BM-MSCs and dendritic cells, since both cell types are semi-adherent and express CD83 [189]. Therefore, immunophenotyping using techniques such as flow cytometry is now considered to be an important approach for defining stromal cell sub-populations [140, 141].

A study by Fazeley *et al.* evaluated the infection of primary human placental fibroblasts with HIV-1 [190]. The isolated cells were classified as mesenchymal due to their expression of vimentin (mesenchymal cells) and lack of factor-VIII (endothelial cells) and CD14 (monocytes/macrophages) expression. MSCs have also been found to reside in different perinatal tissues such as placental tissues (amniotic membrane, chorionic plate, and decidua parietalis) and the umbilical cord [191]. It can thus be postulated that the placental fibroblasts evaluated by Fazeley may have contained a subset of the cells we know today as MSCs. The authors reported that placental fibroblast infection by HIV-1_Lai_ and HIV-1_IIIB_ could only be detected by rescuing virus with susceptible target cells, which is suggestive of latent infection.

Other early studies were also interested in adipose cells as possible targets for HIV-1 [32–34]. These studies however did not analyse MSCs/ASCs per se but evaluated preadipocytes isolated and differentiated from human subcutaneous adipose tissue. The methods used to isolate these preadipocytes [32, 33] are similar to those used to isolate ASCs [133, 134]. Thus, the cells they classified as preadipocytes may have contained a subset of ASCs. Hazan *et al.* isolated preadipocytes from adipose tissue harvested from HIV-1 seronegative donors, and investigated whether these adipose cells supported HIV-1 entry [32]. Primary human preadipocytes exposed to HIV-1 X4- and R5-trophic viruses had low p24 levels. Additionally, analysis of genomic DNA from preadipocytes 10-15 days post HIV-1 exposure resulted in the detection viral DNA specific for the HIV-1 *gag* region. Taken together, preadipocytes appear to be susceptible to HIV-1 infection; however viral integration and expression was not evaluated. The low-level viral production may be indicative of an infectious replication process, but whether this was efficient viral production was not clearly demonstrated. Reassessment of these experiments by the same authors concluded that adipose tissue cannot be infected with HIV-1 *in vitro* since HIV-1 receptor and co-receptor levels did not permit entry [34]. In addition, adipose cells obtained from donor biopsies failed to show high levels of HIV-1 receptor and co-receptor expression, which may be the limiting factor for efficient viral entry. They further propose that once the entry step is bypassed, viral replication can occur efficiently in adipose cells, but that this is likely to be a rare event. Maurin *et al.* performed similar experiments on adipose cells from human subcutaneous adipose tissue [33]. They demonstrated that inefficient HIV-1 replication in adipose cells was due to a lack of activation of adipocyte-signalling pathways. HIV-1 was able to infect human adipocytes *in vitro* and HIV-1 genes were expressed upon stimulation with pro-inflammatory cytokines. It will be important to repeat the experiments performed by Hazan, Maurin and colleagues using cells characterised as ASCs according to the criteria set out by ISCT and IFATS, to determine whether ASCs can indeed be infected with HIV-1 and contribute to the HIV-1 reservoir. Consensus regarding the expression of CD4, CCR5 and CXCR4 on adipose cells (primary culture preadipocytes, differentiated adipocytes, adipose tissue biopsies etc) has not been reached.

### Recent Experiments

More recent *in vitro* HIV-1 infection studies using MSCs phenotypically identified by flow cytometry have generated contrasting results. Gibellini *et al.* found that undifferentiated, vessel wall-derived MSCs display evidence of infection when exposed to classical laboratory HIV-1 strains HIV-1_lIIb_ (X4-tropic) and HIV-1_ada_ (R5-tropic) [101]. HIV-1 proviral DNA for both strains were detectable in vessel wall-derived MSCs by PCR for HIV-1 *gag* (142 bp). Host cell integrated HIV-1 DNA (100 bp) was also detectable by nested *Alu-gag* PCR [101]. In support of this observation, exposure of these vessel wall-derived MSCs to HIV-1 was found to increase apoptosis, which was attenuated following treatment with CD4 antagonists, suggesting possible HIV-1 CD4 receptor utilisation [101]. HIV-1 p24 levels were analysed by ELISA to determine the presence of replicative HIV-1, and were found to be low and decreasing, suggesting possible low-level infection [101].

Cotter *et al.* observed results similar to those of Gibellini *et al*. [117]. They detected HIV-1 products (100 bp) using nested *Alu-gag* PCR which is indicative of viral integration, both in DNA from HIV-1 positive patients and DNA extracted from HVL-exposed BM-MSCs. Exposure of BM-MSCs to HVL HIV-1 positive sera for 72 hrs also altered MSC properties, which was no longer present upon treatment of the cells with antiretroviral drugs or CD4 antagonists. The authors concluded that the effect of HIV-1 on BM-MSCs may be due to infection and integration into the host genome. In subsequent experiments, the authors could not verify a productive infection of HIV-1 exposed BM-MSCs, since viral p24 and Tat proteins were undetectable by ELISA and ICC, respectively. However, the authors could detect Rev, the first protein to be translated in the HIV genome, by ICC. This partial transcription of viral proteins may have been due to a latent infection.

Nazari Shafti *et al.* found that undifferentiated ASCs exposed to low levels of HIV-1 (MOI of 0.1) had negligible p24 levels in the culture supernatant, and expression of Gag and Tat HIV-1 proteins was not evident in ASCs [111]. Productive infection was not detected in HIV-1 exposed ASCs, whereas ASCs that underwent differentiation into cells with hematopoietic attributes, referred to as hematopoietic differentiated (HD) cells, and then exposed to HIV-1, presented with detectable p24 levels, *Gag* and *Tat* mRNA, all indicative of productive infection. However, HD cells derived from ASCs pre-exposed to HIV-1 failed to show evidence of productive infection [111]*.*

## Effect of HIV-1 on MSC Properties

HIV-1 infected individuals display bone and lipid toxicities; however, the exact mechanism underlying these toxicities is not fully understood. Interest has arisen in determining the effects of HIV-1 on MSCs and whether bone and lipid toxicities in HIV-1 positive individuals might result from altered MSC function. MSCs are involved in lipid metabolism and bone mineralisation and can differentiate into adipocytes and osteoblasts *in vitro* [192]. The formation of fat and bone is inversely related [193, 194] and is controlled by an antagonistic balance between peroxisome proliferator-activated receptor gamma (PPARγ, which induces adipogenesis) and the runt-related transcription factor-2 (RUNX-2, which drives osteogenesis) [195]. The effect of various HIV-1 proteins, such as Tat, gp120, p55-gag, Rev and Nef on MSC properties has been investigated and will be discussed in detail below. Table 3 summarises this information and highlights the lack of studies investigating the effects of HIV-1 proteins on ASCs.

**Table 3:** MSC characteristics affected by HIV-1 infection and/or exposure to HIV-1 proteins *in vitro*

MSC source	Treatment	Characteristic	Outcome	Proposed mechanism	Reference
Vessel wall-derived MSCs	HIV-1 strains: HIV-1IIIb HIV-1ada HIV-1 gp120 protein	Apoptosis	Induced apoptosis in sub-confluent MSCs but failed to induce apoptosis during differentiation.	Direct interaction of gp120 and CD4 with the cell membrane led to apoptosis. Blocking gp120 or CD4 reversed the activation of apoptosis.	[101]
		Adipogenic differentiation	Enhanced adipogenesis.	Upregulates expression of *C/EBP β*, *C/EBP δ*, *adipsin* and *PPARγ* mRNA.	
		Endothelial cell differentiation	Inhibited endothelial differentiation.	Downregulates expression of endothelial markers vWF, Flt-1 and KDR (as detected by flow cytometry and RT-qPCR).	
Vessel wall-derived MSCs	HIV-1 Tat protein	Apoptosis	Induced apoptosis in sub-confluent MSCs with high Tat concentration but failed to induce apoptosis during differentiation.	Tat modulates cell proliferation and survival by inducing pro-apoptotic or anti-apoptotic responses that are dependent on Tat concentration.	[196]
		Adipogenic differentiation	Enhanced adipogenesis.	Upregulates expression of *C/EBP β*, *C/EBP δ,* and *PPARγ* mRNA.	
		Endothelial cell differentiation	Inhibited endothelial differentiation.	Downregulates expression of endothelial markers *vWF*, *Flt-1* and *KDR*.	
BM-MSCs	HIV-1 Rev and p55-gag protein	Osteogenic differentiation	Rev: pro-osteogenic effect. p55-gag: decreased osteogenesis.	Rev increases calcium deposition and ALP activity. p55-gag decreases calcium deposition and ALP activity by downregulating BMP-2 and osteocalcin secretion and *RUNX-2* expression*.*	[197]
BM-MSCs	HIV-1 p55-gag and Rev protein	Osteogenic differentiation	p55-gag: reduced overall level of osteogenesis. Rev: increased overall rate of mineralisation.	p55-gag leads to an earlier increase in CTGF levels, RUNX-2 activity and BMP-2 secretion. Rev reduces BMP-2 secretion, RUNX-2 activity, CTGF levels and ALP activity.	[198]
BM-MSCs	HIV-1 Tat and Nef protein	Cell senescence	Reduced proliferative activity and induced senescence.	Results from increased oxidative stress and mitochondrial dysfunction. Tat induces early increase in NF-κB activity and cytokine/ chemokine secretion, and these effects are prevented with an NF-κB inhibitor. Nef induces early inhibition of autophagy, and this effect is reversed with an autophagy inducer.	[199]
		Osteogenic differentiation	Decreased osteogenic differentiation.	Lower level of RUNX-2 protein, decreased *RUNX-2* mRNA expression and decreased level of osteocalcin secretion.	
BM-MSCs	HIV-1 p55-gag protein	Senescence	Inhibited proliferation and induced senescence.	Not reported.	[200]
		Hematopoietic supportive function	Reduced HSC colony forming capability, HSC expansion and p55-gag BM-MSCs did not express TPO and HGF (by RT-qPCR and ELISA) and had decreased expression for Flt3L, SCF, IL-7, and IL-8 cytokines.	BM-MSC senescence and decreased production of cytokines impacts HSC homeostasis.	
BM-MSCs	HIV-1 Tat protein Transfected with HIV-1 *tat* mRNA	Hematopoietic supportive function	Reduced expansion and CFU forming capacity of HSCs co-cultured with BM-MSC. BM-MSCs lost their ability to assist hematopoietic recovery after co-transplantation of HSCs and BM-MSCs *in vivo.* Decreased expression of hematopoietic cytokines (TPO, Flt3L, SCF, IL-7 and IL-8) by BM-MSCs.	Tat protein inhibits hematopoietic support function of BM-MSCs by reducing hematopoietic cytokine expression by BM-MSCs possibly induced by BM-MSC senescence.	[201]
BM-MSCs	HIV-1 gp120 protein	Cell migration	Enhanced cell migration of MSCs in response to SDF-1.	Upregulates CXCR4 (protein and mRNA expression as well as cell surface expression), leading to amplification of the FAK-Paxillin and ERK 1/2 signalling pathways.	[154]
ASCs	HIV-1 Tat and Nef protein	Proliferation and ECM production	Induced profibrotic phenotype. Increased mRNA and protein levels of collagen 1-α1.	Increases secretion of fibronectin and TGF-β1 and expression of the myofibroblast marker, αSMA.	[202]
		Adipogenesis and ECM production	Nef, but not Tat decreases cellular lipid accumulation. Nef induced collagen 1-α2 expression and both HIV-1 proteins induced collagen 6-α1 expression.	Downregulates expression of PPARγ and FABP4 (protein and/or mRNA).	
BM-MSCs	HIV-1 virus HIV-1 Tat protein	Clonogenic ability and developmental potential	Suppressed the proliferation and differentiation of MSC-derived colony formation.	Increases levels of inflammatory cytokines being released.	[100]

Abbreviations: *(BM)-MSCs* (Bone marrow-derived) mesenchymal stromal/stem cells, *ASCs* adipose-derived mesenchymal stromal/stem cells, *HSCs* hematopoietic stem cells, *TPO* thrombopoietin, *Flt3L* Flt-3 ligand, *SCF* stem cell factor. *IL* interleukin, *HGF* hepatocyte growth factor, *BMP-2* bone morphogenic protein-2, *RUNX-2* Runt-related transcription factor 2, *PPARγ* Peroxisome proliferator-activated receptor gamma, *CTGF* Connective tissue growth factor, *ALP* alkaline phosphatase, *vWF* von Willebrand factor, *Flt-1* Fms related receptor tyrosine kinase-1, *KDR* kinase insert domain, *C/EBP β or δ* CCAAT/enhanced-binding protein beta or delta, *FABP4* Fatty acid-binding protein 4, *ERK 1/2* Extracellular signal-regulated kinase 1/2, *FAK* Focal adhesion kinase, *αSMA* alpha smooth muscle actin, *TGF-β1* Transforming growth factor beta 1, *CXCR4* CXC-chemokine receptor 4, *NFκB* Nuclear factor kappa-light-chain-enhancer of activated B cells, *CFU* colony forming unit, *RT-qPCR* reverse transcription quantitative PCR, *gp120* HIV-1 glycoprotein 120

### Viability, Proliferation, And Differentiation Capacity

In an *ex vivo* model, cultured human BM-MSCs were exposed to serum from uninfected donors as well as treatment naïve HIV-1 positive patients with a HVL (100 000-150 000 copies/mL) or a low viral load (LVL; 200-4 000 copies/mL) for 72 hr, before being differentiated into adipogenic or osteogenic lineages [117]. The authors found that exposure to HIV-1 patient sera favoured pro-adipogenic MSC differentiation and gene expression in a viral-load specific manner, and that this was driven by Tat. The effect of HVL sera on MSCs was attenuated by reverse transcriptase-inhibiting compounds and CD4 antagonists.

*In vitro*, HIV-1 exposure suppressed the proliferative, colony-forming and differentiation capacity of BM-MSCs, possibly through increased expression of inflammatory cytokines [100]. The HIV-1 proteins p55-gag and gp120 reduced osteogenesis in human osteoblast cells, while Rev and p55-gag dysregulated osteogenesis in BM-MSCs [197]. In a follow-up study, exposure to Rev increased the overall rate of mineralisation whereas p55-gag had the opposite effect, reducing osteogenesis [198]. The investigators found that p55-gag and Rev could significantly alter MSC osteogenesis by altering key signalling events during the differentiation process (alkaline phosphatase (ALP) activity, calcium deposition, bone morphogenic protein- 2 (BMP-2), RUNX-2, connective tissue growth factor (CTGF)).

In human vessel wall-derived MSCs, HIV-1 and recombinant gp120 [101] and Tat [196] increased MSC apoptosis and regulated their differentiation capacity. Exposure to either gp120 or Tat increased adipogenesis by upregulating PPARγ activity, whereas endothelial differentiation was impaired by downregulating expression of endothelial markers Von Willebrand factor (vWF), Fms related receptor tyrosine kinase-1 (Flt-1) and kinase insert domain (KDR). Tat and Nef also reduced the proliferative activity of human BM-MSCs, promoting senescence and altering osteoblastic differentiation [199]. The investigators explored the mechanism of the Tat and Nef effects and found that Tat induced senescence via NF-κB pathway activation, resulting in oxidative stress, while Nef induced senescence by inhibiting autophagy. The reduced ability of BM-MSCs treated long term with Tat and/or Nef to differentiate into the osteogenic lineage correlated with decreased levels of RUNX-2 protein and mRNA expression, and osteocalcin secretion. Using an HIV-1 mouse model, BM-MSCs isolated from Tg26 HIV-1 transgenic mice showed reduced proliferation, osteogenic differentiation, function (impaired expression of multiple cytokines and chemokines) and therapeutic potential compared to BM-MSCs from healthy mice [203]. *In vitro*, Tat and Nef induced a profibrotic phenotype in proliferating ASCs by upregulating production of fibronectin, TGF-β1, alpha smooth muscle actin (αSMA), collagen 1-α2 and collagen 6-α1 [202]. Nef altered the adipogenic differentiation capacity of ASCs by reducing cellular lipid accumulation and expression of adipogenic markers PPARγ and fatty acid binding protein 4 (FABP4) [202].

### Hematopoietic Supportive Functions

BM-MSCs are known for their hematopoietic supportive functions; however, resident MSCs could be impaired by HIV-1 proteins released by infected cells leading to the well-known cytopenia’s found in HIV-1 patients. It has been suggested that p55-gag and Tat may influence the hematopoietic supportive function of MSCs *in vitro* [200, 201]. HSPCs cultured with BM-MSCs exposed to p55-gag had fewer living cells and fewer colony forming units compared to HSPCs cultured with unexposed BM-MSCs [200]. BM-MSCs treated with p55-gag also showed reduced proliferation and higher senescence rates in culture. These studies postulate that senescence in BM-MSC impaired their ability to support HSPC proliferation and survival. BM-MSCs exposed to Tat displayed a reduced ability to support expansion of HSPCs *in vitro*. *In vivo*, Tat exposed BM-MSCs failed to support hematopoietic reconstitution after co-transplantation with HSPCs in sublethal-dose-irradiated NOD/SCID mice compared to HSPCs transplanted with unexposed BM-MSCs [201].

## Discussion

MSCs reside in the bone marrow and in adipose tissue, both of which are sites of HIV-1 infection [31, 143, 204, 205]. The proximity of MSCs to HIV-1 susceptible cells *in vivo*, their haematopoietic supportive functions, involvement in regulating adipogenesis and osteogenesis, ability to home to sites of inflammation and/or tissue injury, and ability to modulate the immune response, make them a potential cell reservoir for HIV-1. The clinical application of MSCs is gaining momentum and they are being evaluated in clinical trials for musculoskeletal defects, immune system disorders and myocardial infarction, amongst others [206]. There is a need to understand whether MSCs can become infected with and harbour HIV-1 before they can be safely administered clinically. Treatment with MSCs to improve host immune reconstitution outcomes in HIV-1 positive patients treated with HAART who are immune non-responders (INRs), has been proposed [207, 208]. It would be imperative to understand the interaction of MSCs and HIV if we are to treat HIV-1 patients with MSCs. If HIV-1 negatively affects MSC properties and if MSCs can harbour HIV-1, then we cannot expect them to have an effective therapeutic effect.

The experiments described in this review focused mainly on whether MSCs could become infected with HIV-1 and how this affected their characteristics such as proliferation, differentiation capacity and hematopoietic supportive functions. These experiments fell short of being able to prove whether the low productive infection detected in MSCs was indeed due to latent infection. There is a need for additional research focused on specific areas, such as whether (i) MSCs from different sources (bone marrow, adipose tissue etc) express HIV-1 entry receptors and co-receptors at levels sufficiently high enough *in vivo*, *ex vivo* and *in vitro* to allow for active HIV-1 entry into these cells; (ii) MSCs support HIV-1 integration and not only HIV-1 DNA presence; (iii) MSCs allow productive and/or latent infection to occur; and (iv) MSCs can be classified as an additional HIV-1 reservoir by proving the presence and determining the size of this latent reservoir.

The levels of expression of the HIV-1 CD4 receptor and CCR5 and CXCR4 co-receptors are a limiting factor for HIV-1 entry and directly correlate with the level of productive or latent infection [209–211]. Cell surface markers such as sole markers (used to select for or purify MSCs from their *in vivo* environment) and stemness markers (used to identify a subset of MSCs with high fibroblastic colony-forming units (CFU-Fs) and differentiation potential) are not stably expressed on MSCs and vary between MSCs from different sources and within MSC sub-populations [212]. For example, the presence of markers Stro-1, CD271, SSEA-4 and CD146 differs between MSCs from various sources [212]. The expression of CD4, CCR5 and CXCR4 receptors/co-receptors on MSCs is no exception. HIV-1 receptors/co-receptors are detectable at the mRNA and protein levels in MSCs; however, expression levels differ between MSC sub-populations [101, 117, 153–155, 159–161, 194]. It is not yet known whether MSCs consistently express HIV-1 receptors/co-receptors on the cell surface at levels sufficient to allow for HIV-1 infection.

To conclusively confirm the presence or absence of HIV-1 entry receptors and co-receptors on MSCs, it will be imperative to understand the strengths and limitations of the techniques used when interpreting the data. Every method requires adequate controls to be included so that false-positive and false-negative outcomes can be excluded. mRNA expression data on its own is not sufficient and must be related to protein expression on the cell surface. In addition, further analysis is needed to determine whether detected mRNA levels are sufficient for protein synthesis, surface expression and functionality of the receptor. Ultimately mRNA and cell surface receptor expression data will be needed alongside functional experiments where cells are exposed to HIV-1 and infection is evaluated in order to conclude whether MSCs express functional receptors/co-receptors at a level sufficient to allow for HIV-1 entry. MSCs should also be evaluated before and after HIV-1 exposure since cell-surface receptors may only become expressed on the cell surface at detectable levels with specific signalling.

In certain HIV-1 susceptible cell types, interaction with HIV-1 or HIV-1 proteins can cause an up- or downregulation of HIV-1 receptor/co-receptor expression. For instance, CD4^+^ T cells from HIV-1 positive patients show upregulated CCR5 expression, and CD4^+^ and CD8^+^ T cells as well as CD14^+^ monocytes had downregulated CXCR4 expression compared to cells from HIV-1 negative patients [213]. The authors proposed that the upregulation of CCR5 resulted in a favourable environment for infection by R5-tropic HIV-1, the dominant viral strain type during early stages of infection. Similarly, the combined actions of HIV-1 proteins, Nef and Vpu, released post- HIV-1 infection, caused decreased cell-surface expression of CD4 on T cells, resulting in higher levels of infection [214–217]. Decreasing CD4 expression seems counterintuitive since CD4 is required for viral entry; however, it has been shown that high levels of CD4 can interfere with HIV-1 infectivity [214, 215, 218]. Although MSCs do not appear to consistently express HIV-1 receptors/co-receptors, MSCs may become primed to express elevated levels of these receptors, which is conducive to HIV-1 infection upon interaction with the virus [154].

The HIV-1 gp120 protein may be involved in priming MSCs to permit HIV-1 infection. As gp120 is shed from the viral membrane, it accumulates in tissues, such as lymphoid tissues [219]. Its presence induces apoptosis and alters the immune response to the virus, impeding viral clearance in CD34^+^ HSPCs and CD4^+^ T cells [220, 221]. Exposure to gp120 upregulates the expression of CXCR4 on MSCs, elicits apoptosis in sub-confluent MSCs, and alters their *in vitro* differentiation capacity by enhancing adipogenesis and inhibiting endothelial cell differentiation [101, 154]. This suggests that MSCs express HIV-1 receptors/co-receptors on the cell surface at levels that is sufficient to allow for the interaction of gp120 with MSCs and to initiate cell death pathways in these cells [101].

The transcriptome, immunophenotype and differentiation capacity of MSCs changes during *in vitro* culturing, and continues to change with increasing passage number [212, 222]. Additionally, enzymatic detachment of MSCs during cell culture passaging may alter or remove receptors on the cell surface, changing the observed MSC receptor expression profile [164]*.* Thus, the HIV-1 receptor/co-receptor expression profile in MSCs may be the consequence of cell culture conditions and needs to be evaluated *in vivo* or on the initial isolated cell population prior to culturing using flow cytometry or immuno-staining [223]*.* Expression may also vary based on tissue localisation, making it difficult to compare studies using MSCs from different sources [224–227]. To circumnavigate these challenges, MSCs, freshly harvested from ART-receiving or treatment naïve HIV-1 positive patients, should be evaluated for HIV-1 receptor and co-receptor expression prior to culturing. Affan *et al.* used freshly harvested or cultured synovial MSCs from tissue biopsies to confirm differences in phenotype between the cells [228]. They found that the surface marker expression of freshly harvested MSCs was distinct from that of cultured cells, emphasising the need for experiments using freshly harvested cells.

Whether MSCs can also become infected with, and harbour HIV-1 *in vivo*, remains to be clearly established. The viral capsid protein p24 is an early virologic biomarker of HIV-1 infection and is mostly used for diagnostic purposes during acute infection [229]. HIV-1 research *in vitro* uses p24 ELISA to detect productive infection. On the other hand, qVOA in combination with p24 ELISA and LRAs can be used as an indicator of viral reactivation to measure the latent HIV-1 reservoir [230]. The papers discussed in this review reported low and sometimes undetectable levels of p24 in HIV-1 exposed MSCs [32–34, 100–103, 111, 117, 122]. It is however difficult to conclude whether latent infection might account for low and undetectable p24 levels. In some studies, p24 levels in culture supernatant were measured over 4 weeks, while in other studies, levels were only measured at 1- or 2-week time-points [100, 117]. Also, MOIs of between 0.01 to 1 were used for *in vitro* infection studies. This is important since the time required for cells to become infected decreases with increasing MOI [231, 232]. Cells exposed to a lower MOI of 0.01 to 0.1 would have required more time to reach a detectable level of infection [231, 232]. In addition to the MOI, it is difficult to determine whether the virus used for these experiments was infectious and replication-competent, since viral propagation in a laboratory setting may introduce mutations which render HIV-1 defective [233, 234]. Co-culture experiments of HIV-1 exposed BM-derived stromal cells with cells susceptible to HIV-1 infection, such as lymphoid and myeloid cells [102], PHA-treated lymphocytes [103], monocytes [122] and T cells [100], was able to amplify viral levels. This viral amplification suggests that although HIV-1 exposed BM-derived stromal cells did not show productive infection, they may have been latently infected since they were able to infect susceptible cells. A low p24 level may imply that MSCs (i) cannot be efficiently infected with HIV-1; (ii) cannot produce HIV-1 virus or produce defective virus; (iii) harbour latent virus; or that (iv) the virus concentration used to infect MSCs was too low. Future experiments need to be carefully planned and include steps to evaluate the reason for low and undetectable p24 levels.

Integration of HIV-1 proviral DNA into host genomic DNA is an important indicator of infection alongside the measurement of viral p24 production. Integrated HIV-1 DNA may also be an indicator of latency in cells that fail to produce virus unless stimulated with LRAs [235]. To detect the presence of HIV-1 DNA in HIV-1 exposed MSCs, conventional and nested *Alu-gag* PCR techniques were used in several studies cited in this review. Nested *Alu-gag* PCR is a highly specific technique used to detect integrated HIV-1 DNA, unlike conventional PCR which cannot differentiate between DNA that exists freely within the cell and DNA integrated into the host genome. Conventional PCR experiments were utilised to test for the presence of HIV-1 DNA in BM-derived stromal cells [100, 102], vessel wall-derived MSCs [101] and BM-MSCs [117], and amplified HIV-1 gene products were detected in all the MSCs evaluated. However, only 2 studies employed nested *Alu-gag* PCR, and identified the presence of integrated HIV-1 gene products [101, 117]. The presence of integrated HIV-1 gene products in MSCs in addition to low or undetectable levels of p24 found in the above-mentioned experiments, suggests that MSCs may harbour latent HIV-1 [32–34, 101–103, 111, 117, 122, 189]. When designing experiments to detect the presence of integration, researchers should focus on targeting conserved HIV-1 DNA regions which are crucial for HIV-1 replication and survival, such as *gag* and *pol*, to limit the chance of detecting defective HIV-1 provirus [236]. This approach is utilised by the Q4PCR technique, which targets four conserved HIV-1 DNA regions [93].

Although various techniques have been employed to confirm that CD4+ T cells contribute to the HIV-1 reservoir (Table 1), only a limited number of these techniques have been used to investigate MSCs. To unequivocally assess whether MSCs are infectable and contribute to the HIV-1 latent reservoir, in-depth studies investigating HIV-1 integration into MSCs, and the infection state of these cells are needed. Additionally, there is a need to compare the HIV-1 production rates between MSCs and the well described rates seen in T cells. Since MSCs are present in known HIV-1 reservoir sites, there is a possibility that MSCs do not become latently infected themselves, but instead contribute to the persistence of HIV-1 by transmitting the virus to or interacting with latently infected cells. A recent study supports this hypothesis and found that MSCs migrate towards latently infected macrophages and T-helper cells, and thereby increase the latency reactivation potential of these cells as quantified by p24 production [237].

To ensure that results are comparable, future research examining the productive or latent infectability of MSCs should address the need for consistency between HIV-1 strains and MSC sources used. Infection experiments performed to date have utilised different strains and tropisms of HIV-1 [100–103, 111, 117, 122, 123]. A study with 11 different strains of HIV-1 highlighted differences in infectivity between the different strains due to differences in HIV-1 Env membrane stoichiometry which affects entry into host cells [238]. Thus, differences in infectivity observed between experiments in this review may be due to the use of different HIV-1 strains. It will also be important to ensure that the same MSC types are compared in infection experiments. Contrasting results were generated regarding the infectability of fibroblast-like and macrophage-like BM-derived stromal cells using R5- and X4-tropic HIV-1 [102, 103]. Fibroblast-like BM-derived stromal cells showed signs of infection by X4-tropic HIV-1 based on detection of p24; however, these cells showed no evidence of infection by R5-tropic HIV-1. The opposite trend was noted with macrophage-like BM-derived stromal cells, which only demonstrated evidence of infection upon exposure to R5-tropic but not X4-tropic HIV-1. It is likely that the differences in susceptibility of these BM-derived stromal cells to infection may have been due to the nature of the cells used. R5- and X4-tropic HIV-1 are known to predominantly target macrophages and T-cells, respectively, amongst other cell types [239, 240].

Although there is not enough data to confirm whether MSCs represent an additional HIV-1 reservoir, they appear to be affected by HIV-1 and its proteins. The studies discussed in this review have reported that HIV-1 proteins, Tat, Nef, gp120, Rev and p55-gag alter basic biological characteristics of MSCs such as self-renewal, proliferation, viability, senescence, apoptosis, colony forming and *in vitro* differentiation capacity [100, 101, 117, 197–203]. Thus, HIV-1 appears to alter MSC properties *in vitro*, but whether this is the case *in vivo* needs to be evaluated. Differentiation capacity into the adipogenic and osteogenic lineages was also affected by HIV-1 infection and/or HIV-1 proteins [101, 196–199, 202]. It therefore seems plausible that the effects of HIV-1 infection on endogenous MSCs could in part contribute to the lipid and bone toxicities reported in patients. Additional experiments are needed to identify the cause of these changes in MSC properties. Are these changes due to direct infection with HIV-1, interactions with HIV-1 proteins, or from interactions and signalling from other infected cells? MSC therapies in HIV-1 patients may not produce a beneficial effect, which may be due to the deleterious effects of HIV-1 on the cells *in vivo* and not necessarily because they do not work for the specific indications.

## Conclusion

The papers reviewed do not provide enough data to verify whether MSCs can become infected with and harbour HIV-1. Additionally, the effect of HIV-1 and HIV-1 proteins on MSC properties such as self-renewal, proliferation, viability, senescence, apoptosis, colony forming and differentiation capacity were well described *in vitro* but not verified *in vivo*. Although MSCs appear to be capable of expressing HIV-1 entry receptors and co-receptors *in vitro*, there is no consensus on the level of intracellular and extracellular expression. Additionally, the results generated *in vitro* are not necessarily representative of what occurs *in vivo*.

The persistent presence of integrated viral nucleic acids and low levels of p24 in cell culture supernatant suggest that MSCs exposed to HIV-1 may be latently infected. However, due to lack of *in vivo* confirmation, low-level productive infection of MSCs or the possibility of MSCs being refractory to HIV-1 infection *in vivo* cannot be ruled out. Therefore, future experiments should evaluate MSCs directly after isolation from HIV-1 infected individuals instead of MSCs exposed to HIV-1 *in vitro*, to determine the infectability of these cells.

There is a paucity of experiments focusing on MSCs from adipose tissue in the articles evaluated in this review. ASCs may be one of the MSC cell types contributing to the reservoir if they are susceptible to HIV infection. Thus, there is a need to examine whether ASCs can harbour integrated HIV-1 DNA and compare the results to BM-MSCs. The studies described in the review failed to prove unequivocally that MSCs are an additional HIV-1 reservoir. To conclusively determine whether MSCs may indeed serve as an additional reservoir for HIV-1, it will be necessary to employ the techniques mentioned in Table 1, which have successfully proven that CD4^+^ T cells function as a reservoir of latent HIV-1.

## Data Availability

Not applicable
